# 2281. Trends in Antibiotic Prescriptions in U.S. Nursing Homes, 2018-2020

**DOI:** 10.1093/ofid/ofad500.1903

**Published:** 2023-11-27

**Authors:** Quynh Vo, Brandon Dionne, Shira Doron, Farzad Noubary, Becky Briesacher

**Affiliations:** Northeastern University, Needham, Massachusetts; Northeastern University, Needham, Massachusetts; Tufts Medical Center, Boston, Massachusetts; Northeastern University, Needham, Massachusetts; Northeastern University, Needham, Massachusetts

## Abstract

**Background:**

An estimated 25% of U.S. nursing home residents may have an antibiotic resistant infection. Antibiotic resistance is primarily driven by antibiotic use, thus, increasing our understanding of trends in antibiotic use within nursing homes is needed to address this public health threat. Our study examined changes in antibiotic prescriptions among nursing home residents using claims data from January 2018 through December 2020.

**Methods:**

Linked files from Medicare Part D and the Minimum Dataset 3.0 covering years 2018-2020 were used to describe antibiotic use in U.S. nursing homes via nationwide trend analysis. Over two million adults, aged 65 years and older, who were covered by Medicare Part D for the entire duration of their stay were included. Changes in trends by antibiotic class and U.S. region were also assessed.

**Results:**

The majority of antibiotics prescribed in U.S. nursing homes were administered by the oral route (98%), and cephalosporins were the most prescribed class of antibiotics (38.6%). The overall proportion of orally administered antibiotics prescribed across U.S. nursing homes increased from January 2018 through December 2020 by 2.6%. There were 155 DOT/1000 patient days in 2018, 166 in 2019, and an increase to 269 in 2020 for oral antibiotics. Intravenous (IV) antibiotic prescribing increased by 71% during this time but only accounted for 2% of all antibiotic prescriptions. There was 1 DOT/1000 patient days in 2018 and 2019 and 3 DOT/1000 patient days in 2020 for IV antibiotics. Variations in prescribing were seen by U.S. region, with the largest increase in oral antibiotics in the West (4.6% increase); and by antibiotic class, with increases in the use of carbapenems (82%), cephalosporins (11.3%), oxazolidinones (70.1%), and Daptomycin (65.3%). All changes were statistically significant at the 0.05 cutoff level.

**Figure:**
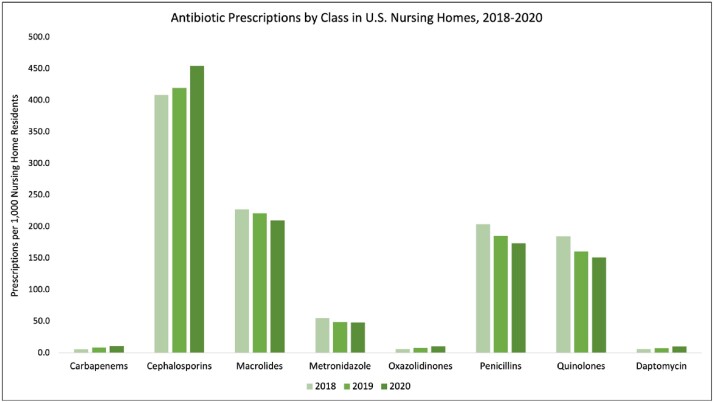

**Figure:**
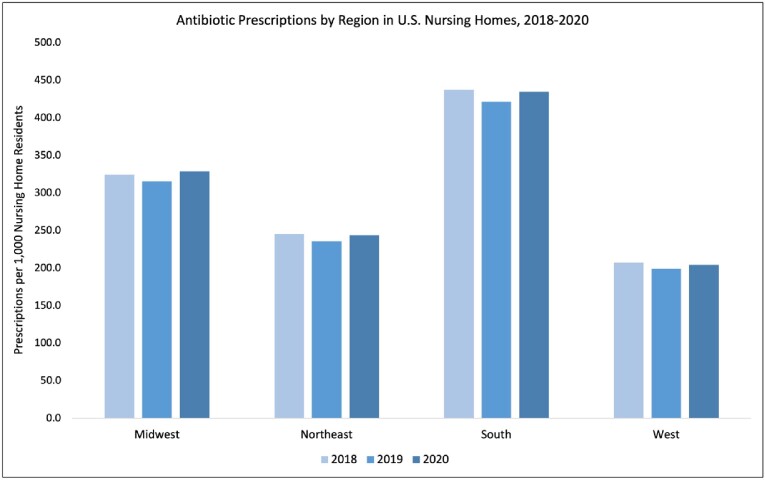

**Conclusion:**

From 2018 through 2019, antibiotic prescribing rates remained relatively unchanged in U.S. nursing homes. However, there were differences by antibiotic class and U.S. region. There was a large increase in the use of both oral and IV antibiotics in 2020, most likely due to confounding factors introduced by the COVID-19 pandemic.

**Disclosures:**

**All Authors**: No reported disclosures

